# Supplementary material for: Discontinuation of oral anticoagulation therapy after successful atrial fibrillation ablation: A systematic review and meta-analysis of prospective studies
Source: PLoS One. 2021 Jun 24;16(6):e0253709. doi: 10.1371/journal.pone.0253709 (PMC8224925; doi:10.1371/journal.pone.0253709)
Supplement: S1 Fig — (PDF) [file pone.0253709.s002.pdf]

S1 Fig. Test for publication bias for the thromboembolic events: (A) Begg's test and (B) Egger' test

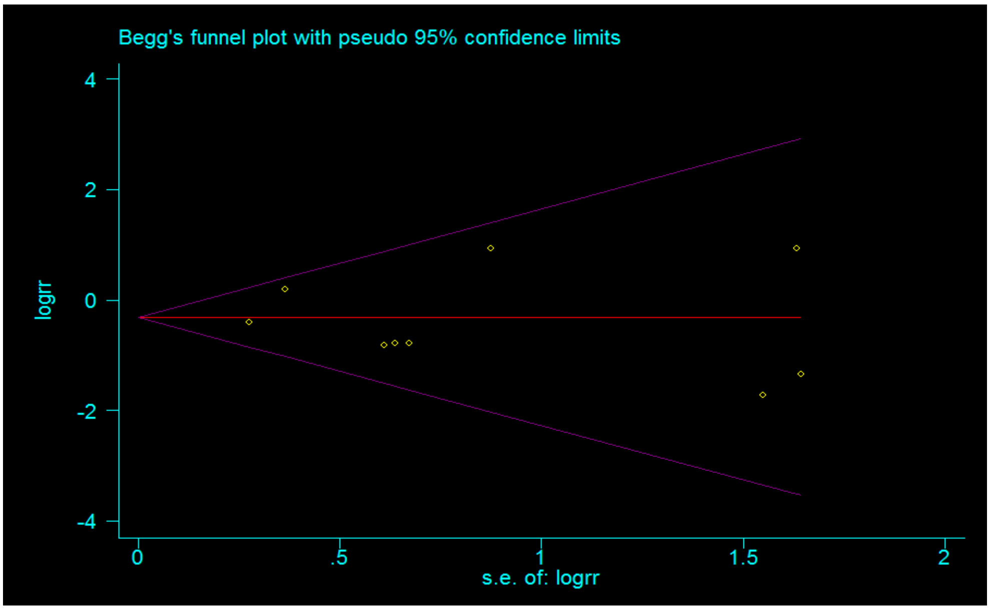


A: Begg's test

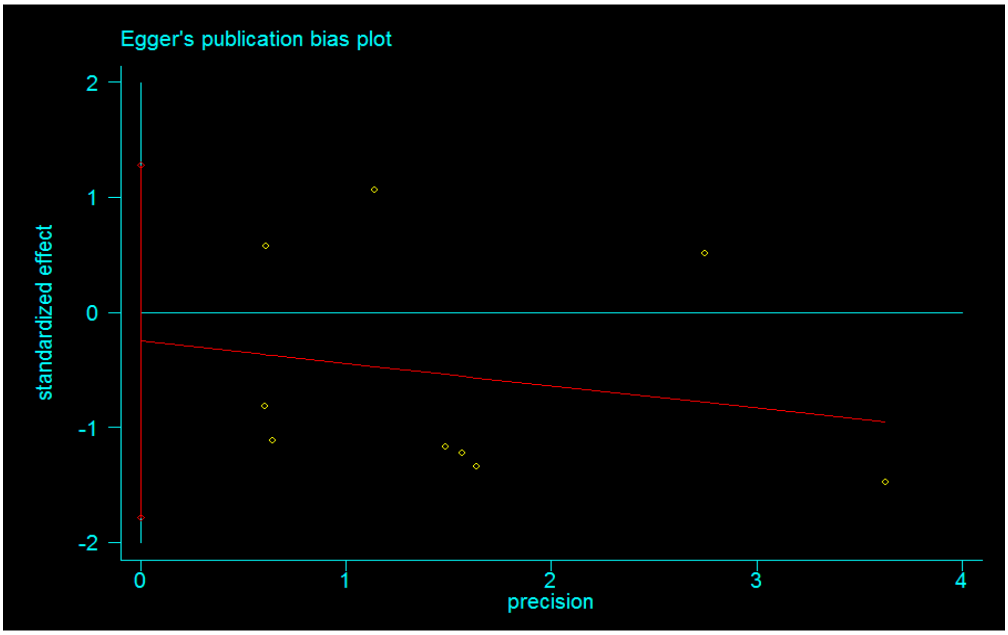


B: Egger's test
