# Supplementary material for: Discontinuation of oral anticoagulation therapy after successful atrial fibrillation ablation: A systematic review and meta-analysis of prospective studies
Source: PLoS One. 2021 Jun 24;16(6):e0253709. doi: 10.1371/journal.pone.0253709 (PMC8224925; doi:10.1371/journal.pone.0253709)
Supplement: S1 Table — (PDF) [file pone.0253709.s003.pdf]

S1 Table. Literature search strategy

The literature search strategy was as follws:
#1 “atrial fibrillation” (title/abstract)
#2 “ablation” (title/abstract)
#3 “anticoagulation” (title/abstract)
#4 “anticoagulant” (title/abstract)
#5 (#3 OR #4)
#6 “dabigatran” (title/abstract)
#7 “apixaban” (title/abstract)
#8 “rivaroxaban” (title/abstract)
#9 “edoxaban” (title/abstract)
#10 (#6 OR #7 OR #8 OR #9)
#11 (#1 AND #2 AND #5 AND #10)
