# Supplementary material for: Discontinuation of oral anticoagulation therapy after successful atrial fibrillation ablation: A systematic review and meta-analysis of prospective studies
Source: PLoS One. 2021 Jun 24;16(6):e0253709. doi: 10.1371/journal.pone.0253709 (PMC8224925; doi:10.1371/journal.pone.0253709)
Supplement: S2 Table — (PDF) [file pone.0253709.s004.pdf]

S2 Table. The Newcastle-Ottawa Scale of individual study

Study	Selection				Comparability	Outcome			Overall score
	Representativeness of the exposed cohort	Selection of the non-exposed cohort	Ascertainment of exposure	Outcome of interest was not present at start of study	Comparability of cohorts on the basis of the design or analysis	Assessment of outcome	Adequate follow-up duration	Adequate follow-up rate	
Oral et al., 2006	★	★	★	★	★★	★	★	★	9
Nademanee et al., 2008	★	★	★	★	★★	★	★	★	9
Hussein et al., 2011	★	★	★	★	★★	★	★	★	9
Hunter et al., 2011	★	★	★	★	–	–	★	–	5
Saad et al., 2011	★	★	★	★	★	★	★	★	8
Winkle et al., 2013	★	★	★	–	★	★	★	★	7
Gaita et al., 2014	★	★	★	★	★	★	★	★	8
Hermida et al., 2020	★	★	★	★	★★	★	★	★	9
Yang et al., 2020	★	★	★	★	★★	★	★	★	9
Yu et al., 2020	★	★	★	★	★★	★	★	★	9
