# Supplementary material for: Discontinuation of oral anticoagulation therapy after successful atrial fibrillation ablation: A systematic review and meta-analysis of prospective studies
Source: PLoS One. 2021 Jun 24;16(6):e0253709. doi: 10.1371/journal.pone.0253709 (PMC8224925; doi:10.1371/journal.pone.0253709)
Supplement: S3 Table — (PDF) [file pone.0253709.s005.pdf]

S3 Table. Incidence of thromboembolism and major bleeding in the included studies

Study and year	Incidence of TE		Incidence of MB		Sample size		Subgroup				
	Off-OAC	On-OAC	Off-OAC	On-OAC	Off-OAC	On-OAC	Ethnicity	Sample size	Mean age	OAC strategy	Follow-up druation
Oral et al., 2006	0	2	0	2	398	357	Non-Asian	<800	≤60 years	W	<3 years
Nademanee et al., 2008	5	5	0	1	434	201	Non-Asian	<800	>60 years	W	<3 years
Hussein et al., 2011	1	0	NA	NA	449	382	Non-Asian	>800	≤60 years	W	>3 years
Hunter et al., 2011	4	5	NA	NA	809	464	Non-Asian	>800	≤60 years	W	>3 years
Saad et al., 2011	0	0	0	3	298	29	Non-Asian	<800	>60 years	W	>3 years
Winkle et al., 2013	0	1	0	9	60	48	Non-Asian	<800	>60 years	W/ DOACs	<3 years
Gaita et al., 2014	5	6	0	7	499	267	Non-Asian	<800	≤60 years	W	>3 years
Hermida et al., 2020	2	4	NA	NA	75	375	Non-Asian	<800	≤60 years	W/ DOACs	<3 years
Yang et al., 2020	34	22	12	9	3149	1363	Asian	>800	>60 years	W/ DOACs	<3 years
Yu et al., 2020	26	11	11	13	989	502	Asian	>800	≤60 years	W/ DOACs	<3 years

DOAC, direct oral anticoagulant ; MB, majoring bleeding; NA, data not available; OAC, oral anticoagulant; TE, thromboembolism; W, warfarin
