# Supplementary material for: Discontinuation of oral anticoagulation therapy after successful atrial fibrillation ablation: A systematic review and meta-analysis of prospective studies
Source: PLoS One. 2021 Jun 24;16(6):e0253709. doi: 10.1371/journal.pone.0253709 (PMC8224925; doi:10.1371/journal.pone.0253709)
Supplement: Attachment — Submitted filename: Response to Reviewers.doc [file pone.0253709.s007.doc]

**Response to reviewers' comments**

**Reviewer #1**

**Comment:** The authors well responded to the comments by the reviewers. The manuscript has been much improved. Finally, please recheck the grammatical especially in the re-edited parts. There are many grammatical errors or insufficient sentences.

**Response**: Thanks for your suggestions. We have rechecked the grammatical errors and insufficient sentences in the full text.

**Reviewer #3**

**Comment:** The present is an interesting paper.

Some issues:

Abstract: kind of studies included should be included (RCTs?; observational studies)

Methods: authors should why they choose Mantel effect

Methods: it should be added if authors pooled studies with analysis corrected at multivariate analysis or not

Results:meta-regression for chads vasc should be performed

Results: warfarin+DOAC is not clear, please write warfarin or DOAC

**Response**: Thanks for your suggestions. According to predefined inclusion criteria and search results, only prospective studies were included in our study. In addition, no published RCT has evaluated off-OAC vs. on-OAC in AF patient undergoing successful ablation.

There are four used methods of meta-analysis for dichotomous outcomes. Our statistical software implements two random-effects methods for dichotomous data: a Mantel-Haenszel method and an inverse-variance method. The difference between the two is subtle. In practice, the difference is likely to be trivial (Cochrane handbook for systematic reviews of interventions, version 5.1.0). There is a common situation in Cochrane reviews, where the Mantel-Haenszel method is generally preferable to the inverse variance method. Therefore, we chose Mantel-Haenszel method in our study.

In our meta-analysis, all pooled analyses were calculated according to studies’ raw data. The relevant data of each included study were presented in the full text and supplementary materials.

We agree with your suggestion to conduct a meta-regression for CHA2DS2-VASc, which is important and significant. However, only 2 studies reported outcomes according to risk stratification. One study reported the thromboembolic and major bleeding events according to the CHA2DS2-VASc score (≤1 and ≥2, respectively), the other study reported outcomes with the incidence rates per 100 person-years according to risk of thromboembolism and major bleeding (including low-, intermediate- and high-risk). These studies reported similar finding, however, it cannot be used for meta-regression. We have contacted the authors by e-mail, however, receiving no reply. Finally, we treated it as a limitation and described it in the manuscript.

We have changed “Warfarin+DOAC” to “warfarin or DOAC” in the revised manuscript.

We would like to thank the referee again for taking the time to review our manuscript.
